# Regional tau deposition measured by [^18^F]THK5317 positron emission tomography is associated to cognition via glucose metabolism in Alzheimer’s disease

**DOI:** 10.1186/s13195-016-0204-z

**Published:** 2016-09-29

**Authors:** Laure Saint-Aubert, Ove Almkvist, Konstantinos Chiotis, Rita Almeida, Anders Wall, Agneta Nordberg

**Affiliations:** 1 Karolinska Institutet, Department NVS, Center for Alzheimer Research, Division of Translational Alzheimer Neurobiology, Novum 5th floor, Huddinge, 141 57 Sweden; 2 Department of Geriatric Medicine, Karolinska University Hospital Huddinge, Stockholm, Sweden; 3 Department of Psychology, Stockholm University, Stockholm, Sweden; 4 Department of Neuroscience, Karolinska Institutet, Stockholm, Sweden; 5 PET Centre, Uppsala University Hospital, Uppsala, Sweden; 6 Nuclear medicine and PET, Department of Surgical Sciences, Uppsala University, Uppsala, Sweden

**Keywords:** Tau imaging, Positron emission tomography (PET), Cognition, Memory, Metabolism

## Abstract

**Background:**

The recent development of tau-specific positron emission tomography (PET) tracers has allowed in vivo quantification of regional tau deposition and offers the opportunity to monitor the progression of tau pathology along with cognitive impairment. In this study, we investigated the relationships of cerebral tau deposition ([^18^F]THK5317-PET) and metabolism ([^18^F]FDG-PET) with concomitant cognitive function in patients with probable Alzheimer’s disease (AD).

**Methods:**

Nine patients diagnosed with AD dementia and 11 with prodromal AD (mild cognitive impairment, amyloid-positive on [^11^C]PiB-PET) were included in this study. All patients underwent PET scans using each tracer, as well as episodic memory and global cognition assessment. Linear models were used to investigate the association of regional [^18^F]THK5317 retention and [^18^F]FDG uptake with cognition. The possible mediating effect of local metabolism on the relationship between tau deposition and cognitive performance was investigated using mediation analyses.

**Results:**

Significant negative associations were found between [^18^F]THK5317 regional retention, mainly in temporal regions, and both episodic memory and global cognition. Significant positive associations were found between [^18^F]FDG regional uptake and cognition. The association of [^18^F]FDG with global cognition was regionally more extensive than that of [^18^F]THK5317, while the opposite was observed with episodic memory, suggesting that [^18^F]THK5317 retention might be more sensitive than [^18^F]FDG regional uptake to early cognitive impairment. Finally, [^18^F]FDG uptake had a mediating effect on the relationship between [^18^F]THK5317 retention in temporal regions and global cognition.

**Conclusions:**

These findings suggest a mediating role for local glucose metabolism in the observed association between in vivo tau deposition and concomitant cognitive impairment in AD.

**Electronic supplementary material:**

The online version of this article (doi:10.1186/s13195-016-0204-z) contains supplementary material, which is available to authorized users.

## Background

Alzheimer’s disease (AD) is characterized by the cerebral accumulation of amyloid-beta (Aβ) into plaques and hyperphosphorylated tau into neurofibrillary tangles (NFT), as well as synaptic and neuronal loss. These different features exhibit distinct patterns of progression along the time course of the disease. Aβ pathological deposition spreads early throughout the neocortex, precedes by many years the onset of clinical symptoms [1], and is not – or is only poorly – associated with markers of atrophy and hypometabolism [2]. The latter two, supposedly more downstream processes, better relate to clinical symptoms [3, 4] and have a tight relationship with the stereotypical regional distribution of NFT pathology [5, 6]. The general thesis is that the intra-neuronal accumulation of tau disrupts the neuronal activity, before leading to the destruction of the affected nerve cells and ultimately to cognitive decline. Autopsy studies have, indeed, confirmed a relationship between NFT and cognitive impairment [7, 8]. However, the relationship between the tau lesions and early cognitive impairment currently remains elusive, as postmortem data are mostly obtained when severe impairment has occurred [8].

So far, only cerebrospinal fluid (CSF) sampling has been able to provide insight into in vivo tau changes in the brains of patients and to relate these changes to concomitant symptoms. However, conflicting results have been reported regarding the relationship between CSF tau biomarkers and NFT pathology [9–12], limiting the validity of such biomarkers in reflecting the pathological progression. Furthermore, CSF biomarkers cannot provide information about the spatial distribution of lesions.

The recent development of tau-specific positron emission tomography (PET) tracers now allows in vivo quantification of the regional distribution of tau deposition (for reviews, see Dani et al. [13] and Villemagne et al. [14]) and offers the opportunity to monitor the progression of tau pathology along with other markers, including cognitive decline [15]. The tracer [^18^F]THK5317 (also known as (*S*)-[^18^F]THK5117) has high target specificity and selectivity [16] and favorable pharmacokinetic properties for non-invasive imaging of tau pathology [17], with good discrimination of AD patients from healthy controls in in vivo PET studies [18, 19].

Theoretical models of AD pathophysiology suggest a sequential association of events in which tau pathology would precede neuronal dysfunction (as identified by [^18^F]FDG PET) that would further affect downstream cognitive processes [20]. Cerebral metabolic disturbances may then play the intermediate between tau deposition and cognitive impairment. In this study, we investigated the relationship between in vivo regional tau deposition (using [^18^F]THK5317 PET) and cognitive performance in patients with probable AD. In addition, we investigated the possible mediation role of cerebral metabolism (using [^18^F]FDG PET) in this association.

## Methods

### Participants

All patients included in this study were enrolled in a larger research study [18], for which they underwent [^18^F]THK5317, [^18^F]FDG, and [^11^C]PiB PET imaging. The patients were referred to the Memory Clinic at the Department of Geriatric Medicine, Karolinska University Hospital (Stockholm, Sweden), because of memory complaint. They underwent thorough clinical investigation including medical history, apolipoprotein E (ApoE) genotyping, neuropsychological assessment, and structural magnetic resonance imaging (MRI). Patients diagnosed with AD dementia fulfilled the revised research criteria for typical probable AD [21], while patients diagnosed with mild cognitive impairment (MCI) met the Petersen criteria [22]. The diagnosis was made by consensus of an expert committee.

Only patients with an amyloid-positive [^11^C]PiB PET scan (see the “PET image pre-processing” section) [23] were included in this study, as they are the most likely to be on the AD pathway. This resulted in 11 patients with amnestic MCI – subsequently referred to as prodromal AD patients [21] – and nine patients diagnosed with AD dementia.

All patients and their caregivers provided written informed consent prior to research investigations, which were conducted according to the Declaration of Helsinki and subsequent revisions. The research project was approved by the regional Human Ethics Committee in Stockholm, as well as by the Radiation Safety Committee of Uppsala University Hospital, Sweden.

### Neuropsychological assessment

Global cognitive performance was assessed using two measures: the mini mental state examination (MMSE) and the full-scale intelligence quotient (FSIQ), which is based on five subtests from the revised Weschler adult intelligence scale (Similarities, Information, Block Design, Digit Span, and Digit Symbol).

Episodic memory performance was assessed using three tests: learning and delayed recall from the Rey auditory verbal learning (RAVL) test, and delayed recall from the Rey-Osterrieth complex figure (Rey) test. In summary, five scores were considered: MMSE and FSIQ (global cognition), RAVL learning, RAVL delayed recall, and Rey delayed recall (episodic memory).

### Image acquisition

All patients underwent a three-dimensional T1-weighted (3D T1W) MRI sequence, 60-min [^18^F]THK5317 and [^11^C]PiB dynamic PET scans to image tau and fibrillar Aβ depositions, respectively, and a 15-min [^18^F]FDG static PET scan to image glucose metabolism. The [^18^F]THK5317 and [^11^C]PiB PET scans were acquired on an ECAT EXACT HR+ scanner (Siemens/CTI) or a Discovery ST PET/CT scanner (GE) at the Uppsala PET Centre (Uppsala, Sweden), after intravenous injection of 212 ± 42 MBq and 253 ± 69 MBq of the tracers, respectively. The [^18^F]FDG PET scans were acquired on a Biograph mCT PET/CT scanner (Siemens) at the Department of Nuclear Medicine, Karolinska University Hospital Huddinge (Stockholm, Sweden), with a 15-min static run, 30 min after injection of 3 MBq/kg. All acquisitions were reconstructed using ordered subset expectation maximization.

### Regions of interest

Fifty bilateral regions of interest (ROIs), excluding the subcortical nuclei, were obtained from the pre-existing Harvard-Oxford structural atlas (FSL software, The University of Oxford) for regional quantification analyses. For each participant, the T1W MRI image was segmented into gray and white matter tissue maps using SPM8 software and the inverse non-linear transformation resulting from this segmentation was used to warp the atlas into each individual’s native T1W space. An inclusive binary gray matter mask was subsequently applied to the resulting atlas to obtain individual gray matter atlases.

### PET image pre-processing

Individual dynamic [^18^F]THK5317 PET images were co-registered onto the individual T1W images with PMOD v.3.5 software (PMOD Technologies Ltd., Adliswil, Switzerland). In order to minimize a possible spill-over effect on the signal from white matter, MRI-based partial volume correction based on individual T1 was applied to the dynamic [^18^F]THK5317 PET images, using the Muller-Gartner method [24] as implemented in PMOD v.3.5 software. The reference Logan graphical method was then applied to the corrected images over the 30 to 60-min scan interval, with cerebellar gray matter as a reference [17], in order to create distribution volume ratio (DVR) images for each participant.

Summed [^11^C]PiB PET (40–60 min) and [^18^F]FDG PET (30–45 min) images from patients were co-registered onto their individual T1W MRI images using SPM8 and standardized uptake value ratio (SUVR) images were created with reference to the cerebellar gray matter for [^11^C]PiB and the pons for [^18^F]FDG. An SUVR threshold of 1.41 for amyloid positivity was applied to [^11^C]PiB PET data [23].

### Statistical analysis

Group differences between patients with prodromal AD (i.e. MCI, PiB-positive) and patients with AD dementia were assessed using the Chi-square test for discrete variables and the Mann–Whitney test for continuous variables.

Linear regression models were performed to investigate the association of [^18^F]THK5317, [^18^F]FDG, and [^11^C]PIB regional retention with cognitive performance in patients with AD (both prodromal AD and AD dementia). These models were performed using the Linear Model (LM) R package. Graphical representations of significant linear associations were obtained with the ggplot2 package v.1.0.1, as implemented in R.

All statistical analyses were performed using the R v.3.1.0 software (The R Foundation for Statistical Computing, http://www.r-project.org/). The significance level for all statistical tests and models was set at *p* < 0.05.

#### Association of [^18^F]THK5317 and [^18^F]FDG regional retention with cognitive performance in probable Alzheimer’s disease

The first linear regression model determined the association between cognitive performance and [^18^F]THK5317 retention after adjusting only for the delay between assessment times. Because age may have an effect on cognition and tau deposition [25, 26], a second model was used to determine the association between cognitive performance and [^18^F]THK5317 retention with adjustment for both delay and age. The *p* value, t-value and the standardized β coefficient (further referred to as “β”) associated with the PET tracers’ regional retention were obtained for each model, as well as the Akaike information criterion (AIC). The AIC is a measure of the relative quality of statistical models, allowing determining among several statistical models which model is “best.” The different linear models were compared using ANOVA. The false discovery rate (FDR) correction for multiple comparisons was applied, with both corrected and uncorrected results reported.

The same linear regression models and same corrections were used to investigate the association of both [^18^F]FDG uptake and [^11^C]PIB retention with cognitive performance in our sample.

In summary, the following linear models were tested:

- Model 1: Cognition = PET tracers’ regional retention + delay + intercept;
- Model 2: Cognition = PET tracers’ regional retention + delay + age + intercept.

#### Mediation analyses

The possible mediating effect of local (i.e. in the same ROI) hypometabolism on the relationship between tau deposition and cognitive performance was investigated using exploratory analyses. In its simpler form, a mediation analysis explores the hypothesis that the relationship between an independent variable X and a dependent variable Y is mediated by a third variable M. This hypothesis explores causal relationships and assumes that the independent variable would have an indirect effect on the dependent variable through the mediator, i.e. X affects M, which in turn affects Y. Mediation analyses were performed using regional [^18^F]THK5317 retention (adjusted for age and delay) as the independent variable, regional [^18^F]FDG uptake (adjusted for age and delay) as the mediator, and cognitive performance as the dependent variable, when the following conditions were met in the first linear model: (1) cognitive performance was significantly associated with [^18^F]FDG regional uptake (without correcting for multiple comparisons); and (2) [^18^F]FDG uptake was also associated – as measured by linear regression – with [^18^F]THK5317 retention in the same region. The average causal mediation effect (ACME) and the average direct effect (ADE) were obtained, using the mediation R package, and assessed for significance based on non-parametric bootstrapping (5000 simulations, *p* < 0.05) for all mediation analyses.

## Results

The demographics and cognitive performance results for the patient groups are described in Table 1. The median [interquartile range] delay period in days between cognitive assessment and [^18^F]THK5317, [^18^F]FDG, and [^11^C]PiB PET scans was 71.5 [73.5], 59.5 [163.5], and 70.5 [79.8], respectively.

**Table 1:** Summary of group demographics and neuropsychological assessment of cognitive domains

	MCI PiB-positive (prodromal AD)	AD dementia
Number	11	9
Demographics		
Age at testing (years)	69.7 ± 6.7	67.3 ± 7.1
Education	12.5 ± 3.3	13.3 ± 2.7
Gender (M/F)	5/6	2/7
ApoE ε3ε3/ε3ε4/ε4ε4	4/1/5	1/5/3
Cognitive functions		
Global cognition		
MMSE	27.7 ± 3.0	22.6 ± 3.3**
FSIQ	92.6 ± 12.2	69.2 ± 21.6*
(z-scores)	−0.8 ± 0.9	−2.6 ± 1.6
Episodic memory		
RAVL, learning	34.7 ± 10.0	20.9 ± 5.7**
(z-scores)	−1.2 ± 1.0	−2.5 ± 0.5
RAVL, delayed recall	3.8 ± 3.0	1.7 ± 2.2
(z-scores)	−1.8 ± 0.9	−2.4 ± 0.6
Rey, delayed recall	12.8 ± 8.1	1.2 ± 1.9**
(z-scores)	−1.0 ± 1.2	−2.7 ± 0.3

Performance is expressed as mean ± standard deviation to the mean. Z-scores were calculated in comparison to a normative population

Statistically significant differences between groups are displayed as *p < 0.05; **p < 0.01

*AD* Alzheimer’s disease, *ApoE* apolipoprotein E, *F* female, *FSIQ* full-scale intelligence quotient, *M* male, *MCI* mild cognitive impairment, *MMSE* mini mental state examination, *PiB* Pittsburgh Compound B, *RAVL* Rey auditory verbal learning test, *Rey* Rey-Osterrieth complex figure test

### Association between [^18^F]THK5317 regional retention and cognitive performance in probable AD

**Fig. 1:**
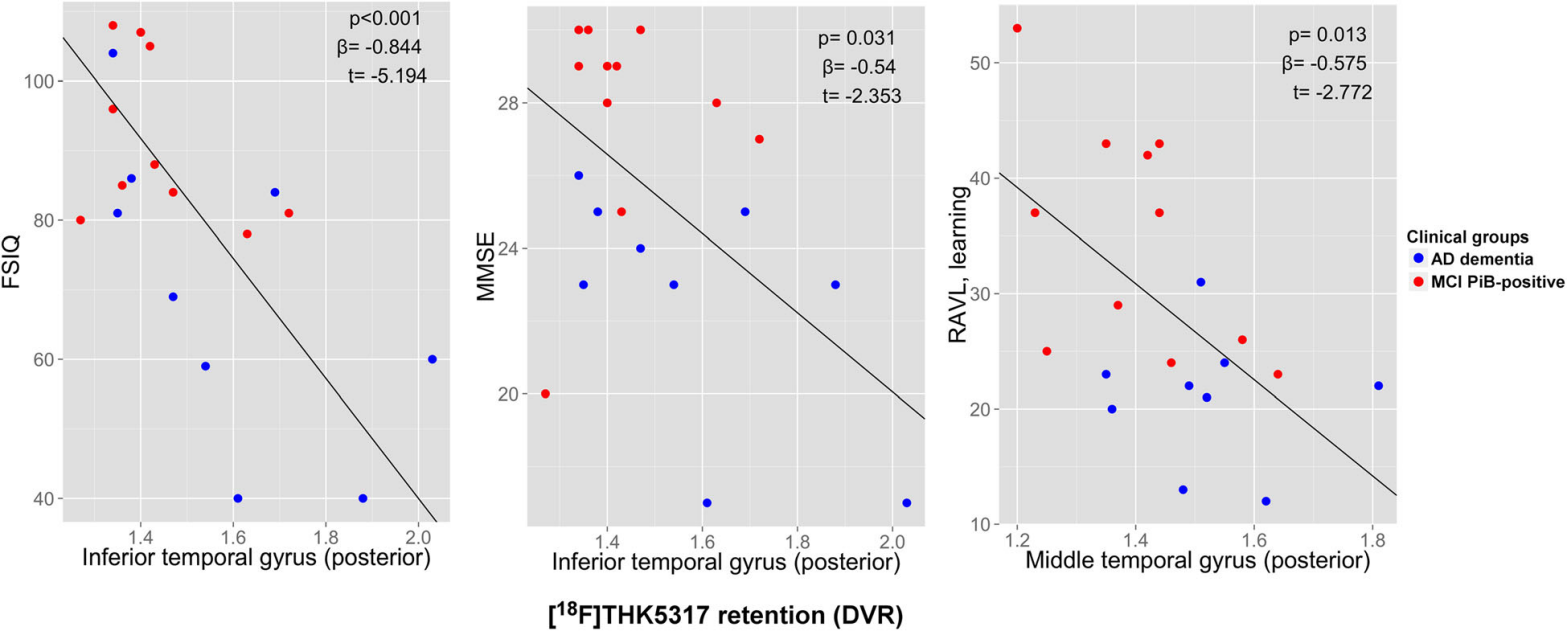
Significant associations between regional [^18^F]THK5317 retention (DVR) and cognition in AD patients, after adjusting for delay. Prodromal AD patients are displayed in *red* and AD dementia patients in *blue*. The *graphs* also show the *p* and t values and the standardized β coefficient associated with regional [^18^F]THK5317 retention in each model. *FSIQ* full-scale intelligence quotient, *MCI* mild cognitive impairment, *MMSE* mini mental state examination, *RAVL* Rey auditory verbal learning test

**Fig. 2:**
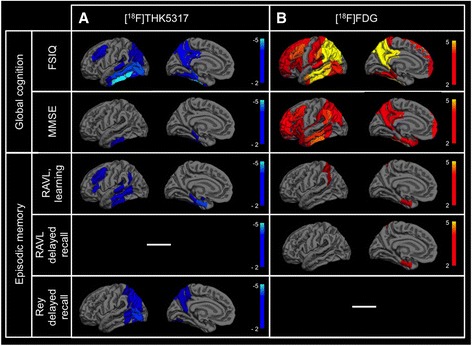
Cortical regions of interest where PET tracer retention was significantly associated with cognition in AD patients, after adjusting for delay (not corrected for multiple comparisons). Linear models assessed the association between cognitive performance using five different tests (each *row*) and (**a**). [^18^F]THK5317 retention (DVR) or (**b**). [^18^F]FDG uptake (SUVR). Regions are defined according to the Harvard-Oxford atlas. T-values for significant negative associations are displayed with a *blue color scale* and T-values for significant positive associations are displayed with a *red color scale. FSIQ* full-scale intelligence quotient, *MMSE* mini mental state examination, *RAVL* Rey auditory verbal learning test, *Rey* Rey-Osterrieth complex figure test

In the first linear model, adjusting for delay between assessments, significant negative associations were found between [^18^F]THK5317 regional retention and global cognition as measured by FSIQ performance. The cortical regions involved in this association were mainly temporal, including the posterior part of the parahippocampal gyrus, the fusiform gyrus, and the inferior, middle, and superior temporal gyri (Fig. 1), with occipital regions and the middle frontal gyrus, the posterior cingulate gyrus, the parietal operculum, and the precuneus also involved. [^18^F]THK5317 retention in the posterior parahippocampal gyrus and the inferior temporal gyrus (posterior part) was also negatively associated with the MMSE score. Significant negative associations were found between [^18^F]THK5317 regional retention and episodic memory performance, as assessed by the RAVL learning and Rey delayed recall tests. These associations mainly concerned temporo-parieto-occipital regions (Fig. 2a and Additional file 1).

After correcting for multiple comparisons, the negative association between [^18^F]THK5317 retention in the inferior temporal gyrus and the FSIQ score remained significant (see Additional file 1).

### Association between [^18^F]FDG regional uptake and cognitive performance in probable AD

Significant positive associations were found between [^18^F]FDG regional uptake and global cognition – using both FSIQ and MMSE scores, after adjusting for delay. These associations were found in a large number of cortical regions that were widespread throughout the cortex. Several associations survived correction for multiple comparisons (Fig. 2b and Additional file 1). A few significant positive associations were found between [^18^F]FDG regional uptake and episodic memory performance, as measured by the RAVL subtests, but they did not survive multiple comparisons correction.

### Association between [^11^C]PiB regional retention and cognitive performance in probable AD

The association between [^11^C]PiB regional retention and cognitive performance was also assessed adjusting for delay between assessments. No significant association was found, either with or without correction for multiple comparisons, between cognition and [^11^C]PiB retention in any region of interest.

### Effect of age

Adding age as a covariate significantly improved the linear models for a few associations between [^18^F]THK5317 regional retention and cognition, but not for associations between [^18^F]FDG uptake and cognition (Table 2). The overall patterns of association with cognitive performance did not change for either [^18^F]THK5317 or [^18^F]FDG (see Additional file 2).

**Table 2:** Akaike information criterion indices for significant linear regression models

	FSIQ		MMSE		RAVL, learning		RAVL, delayed recall		Rey, delayed recall	
Regional retention	Model 1	Model 2	Model 1	Model 2	Model 1	Model 2	Model 1	Model 2	Model 1	Model 2
[^18^F]THK5317										
Amygdala	184	183	119	119	151	149	101	93*	142	138*
Parahippocampal gyrus (post.)	173	170*	114	114	152	154	103	103	138	134*
Parahippocampal gyrus (ant.)	181	178*	116	115	147	145	102	98*	140	133*
Angular gyrus	178	177	118	120	153	155	105	105	137	133*

Model 1: Cognition = [^18^F]THK5317 regional retention + delay + intercept; Model 2: Cognition = [^18^F]THK5317 regional retention + delay + age + intercept. Only associations with significant differences (*) between models (using ANOVA) are displayed

*Ant* anterior part, *FSIQ* full-scale intelligence quotient, *MMSE* mini mental state examination, *post* posterior part, *RAVL* Rey auditory verbal learning test, *Rey* Rey-Osterrieth complex figure test

### Mediation analyses

Linear regression analysis found significant negative associations between [^18^F]FDG uptake and [^18^F]THK5317 retention in the posterior inferior temporal gyrus (t = −2.741; *p* = 0.013), the posterior middle temporal gyrus (t = −2.253; *p* = 0.037), and the occipital fusiform gyrus (t = −2.176; *p* = 0.043).

**Fig. 3:**
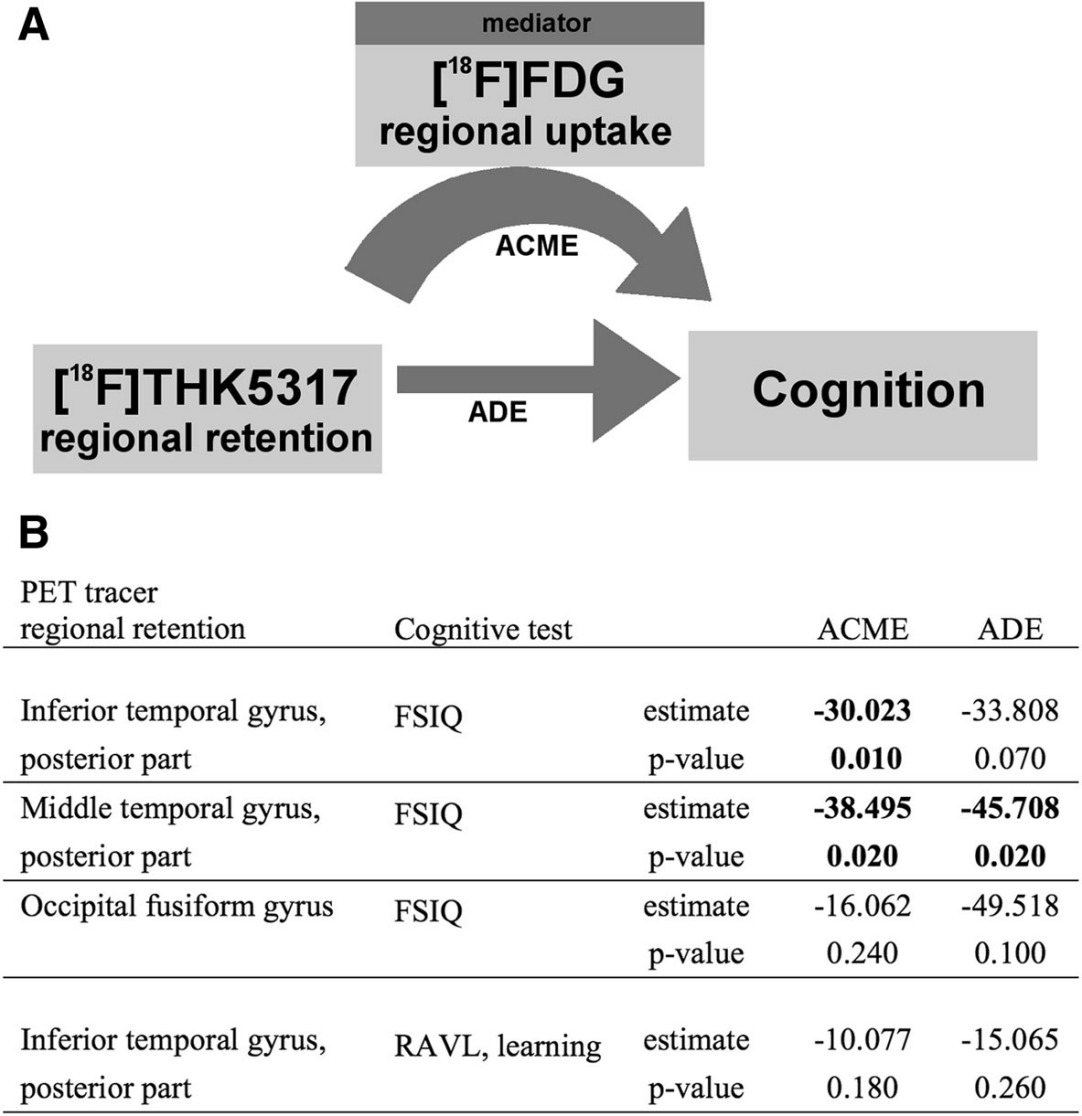
Causal mediation analyses. The model, presented in (**a**), assessed the effect of [^18^F]FDG regional uptake (mediator) on the relationship between [^18^F]THK5317 retention (independent variable) in the same region and cognitive performance (dependent variable). (**b**) Results for the four associations tested. Significant results are displayed in *bold. ACME* average causal mediation effect, *ADE* average direct effect, *FSIQ* full-scale intelligence quotient, *PET* positron emission tomography, *RAVL* Rey auditory verbal learning test

In order to investigate the possible mediating effect for hypometabolism in these three regions in the relationship between local [^18^F]THK5317 regional retention and cognition, mediation analyses were performed using regional [^18^F]FDG uptake as mediator. Mediation analyses were applicable for four associations between cognition and PET regional uptake (Fig. 3). The mediating effect (ACME) of [^18^F]FDG was significant in both the posterior inferior temporal and the posterior middle temporal gyri, while no mediating effect was found in the occipital fusiform gyrus, for the association with FSIQ. No mediating effect was found in the posterior inferior temporal gyrus, for the association with RAVL learning score.

## Discussion

### [^18^F]THK5317 regional retention is associated with cognition

Quantitative assessment of in vivo tau deposition, as measured by [^18^F]THK5317 retention, in the brains of AD patients at different stages of AD revealed a significant association with cognition. To our knowledge, this is the first study investigating the relationship between regional [^18^F]THK5317 retention and concomitant cognition in vivo. [^18^F]THK5317 retention was negatively associated with both global cognition and episodic memory. The cortical regions involved in this association were in agreement with those primarily affected by tau pathology in AD [27, 28]. These findings support the hypothesis that regional tau deposition in AD is closely related to cognitive impairment [8].

A few negative associations between [^18^F]THK5317 retention and cognition were also found in the parietal and frontal cortices. While the medial temporal lobe is among the first to be affected by NFT, the neocortex is thought to be affected by NFT and neuropil threads at later stages [27]. The presence of tau lesions in neocortical regions can, however, be observed in some neuronal layers from Braak stage III [29]. In the light of the reported regional distribution of [^18^F]THK5317 retention in AD patients in comparison to controls [18] and previous in vitro work showing the good binding affinity of the tracer to tau deposits [16], we hypothesize that our findings illustrate the presence of tau pathology in neocortical regions that is related to cognitive impairment. Interestingly, while the association between [^18^F]THK5317 retention and the FSIQ score was seen in several ROIs, the association with the MMSE score was only seen in the inferior temporal gyrus – a finding recently reported with another tau tracer [30] – and the parahippocampal gyrus. These results suggest that global cognitive impairment as measured by composite cognitive scores, such as FSIQ, would better reflect pathological tau progression from the early clinical stages than MMSE.

### Distinctive pattern of association between cognition and [^18^F]FDG regional uptake

Cerebral metabolism, as measured by [^18^F]FDG uptake, was positively associated with both global cognition and episodic memory. The pattern of association with global cognition was much broader than that observed with [^18^F]THK5317, involving frontal, temporal, and parietal regions. This is consistent with previous studies that showed metabolic disturbances beyond the temporo-parietal regions correlating with non-memory performance [31, 32] and suggests that [^18^F]FDG uptake may be more selective than [^18^F]THK5317 retention with respect to global cognitive impairment. [^18^F]FDG uptake was positively associated with episodic memory in restricted areas of the medial temporal and parietal cortices [33], a regional pattern of association less broad than that seen with [^18^F]THK5317. This is consistent with the classical association pattern of hypometabolism with memory impairment in AD [4, 34]. The limited extent of this association, in comparison with that of [^18^F]THK5317, suggests that [^18^F]THK5317 retention might be more selective than [^18^F]FDG uptake with respect to early cognitive (i.e. episodic memory) impairment.

Of note, no significant association between regional [^11^C]PiB retention and cognition was found in our sample. Although we acknowledge that [^11^C]PiB PET retention being part of the inclusion criteria is a clear source of bias in its interpretation, this finding highlights the differential relationships of amyloid and tau deposits with symptoms in the same population of patients with probable AD.

### [^18^F]FDG regional uptake mediates the relationship between [^18^F]THK5317 temporal retention and global cognition

Hypothetical models of biomarker progression in AD suggest that the increase in cerebral tau pathology would precede metabolic disturbances, which in turn would precede the onset of symptoms [35]. The relationships among these three have, however, remained elusive. When we investigated the relationship between both tracers and cognition in these patients, we found evidence of a mediating role for local cerebral glucose metabolism in the relationship between tau deposition and global cognition – as measured by the FSIQ score. This mediating effect was observed in two temporal regions: the inferior and middle temporal gyri. This illustrates the existence of potential intermediate factors between pathological lesions in neurons and clinical symptoms and suggests a regional causal sequence where local accumulation of tau lesions triggers neuronal dysfunction, which in turn leads to cognitive impairment. This intermediate role for glucose metabolism between lesions and symptoms has also been suggested in a recent study exploring the mediating effect of metabolism in the relationship between CSF biomarkers and cognition [36]. The authors suggested that the tau-related burden precedes neurometabolic dysfunction, which results in subsequent cognitive impairment. Although the role of tau deposits, and especially of NFT, in AD is not well understood, their presence intra-cellularly is likely to contribute to synaptic dysfunction [37] or neuronal activity changes [38]. These can be indirectly measured in vivo by assessing regional glucose metabolism. Our findings suggest that tau pathology in temporal regions would interfere with local neuronal activity, which would further disrupt neuronal circuitry and lead to cognitive impairment. We hypothesize that the accumulation of tau would first trigger minor neuronal disturbance leading to early (episodic memory) cognitive impairment and then larger disruption of neuronal functions as reflected by cerebral glucose hypometabolism leading to more global cognitive impairment.

### Other factors likely to contribute to the association between tau and cognition

Normal aging has already been found to play a significant role in cognition [26] as well as in tau deposition [25] and cerebral glucose metabolism [39] in specific cerebral regions. As a consequence, we adjusted all variables relevant to age in our linear models, which improved the results for several associations. Several other factors that could not be accounted for here, such as neuroinflammation and genetic factors, may also play a key role in the relationship between tau deposition and cognition [40]. A large body of studies suggest that tau pathology is necessary but not sufficient to trigger neuronal dysfunction. Tau itself is seen by some as a key mediator of amyloid-induced toxicity for neuronal activity (see the review by Liao et al. [41]). We acknowledge that our study only reflects one aspect of the pathological processes. Furthermore, it is likely that tau lesions in one region could trigger metabolic dysfunction in remote regions, leading to further cognitive impairment. The limited sample size also restricted our investigations to linear models, while non-linear models might better reflect the relationships between the variables over time. This deserves to be addressed in further studies with larger sample sizes. In addition, because of the large number of regions included in the analyses, only a few linear models showing a significant association between regional PET uptake and cognition survived after correction for multiple comparisons. To minimize the number of comparisons, only bilateral ROIs were investigated. However, hemispheric differences could be expected, in view of the known specialization of each hemisphere for memory subtypes.

## Conclusions

Our study indicates that the cerebral distribution of tau deposits assessed in vivo using the [^18^F]THK5317 PET tau tracer is strongly associated with cognitive impairment in patients with probable AD. The second major finding is that tau PET imaging could be more selective for early cognitive (i.e. episodic memory) impairment than metabolic changes. Finally, evidence from this study suggests that metabolic dysfunction could, at least partly, play a mediating role in the association between tau pathology and cognitive impairment in AD patients.

## Additional files

Additional file 1: Association of [^18^F]THK5317 distribution volume ratio (DVR) and [^18^F]FDG standardized uptake value ratio (SUVR) regional retention with cognitive performance, as determined by linear regression modeling, after adjusting for delay between assessments. Only significant *p* values (<0.05) are displayed. Values in bold indicate associations that survived false discovery rate (FDR) correction for multiple comparisons. *β* standardized β coefficient, *FSIQ* full-scale intelligence quotient, *MMSE* mini mental state examination, *RAVL* Rey auditory verbal learning test, *Rey* Rey-Osterrieth complex figure test. (DOC 186 kb) Additional file 2: Cortical regions of interest where PET tracer retention was significantly associated with cognition in AD patients, after adjusting for delay and age (not corrected for multiple comparisons). Linear models assessed the association between cognitive performance using five different tests (each row) and (**A**). [^18^F]THK5317 retention (DVR) or (**B**). [^18^F]FDG uptake (SUVR). Regions are defined according to the Harvard-Oxford atlas. T-values for significant negative associations are displayed with a *blue* color scale, and T-values for significant positive associations are displayed with a *red* color scale. *FSIQ* full-scale intelligence quotient, *MMSE* mini mental state examination, *RAVL* Rey auditory verbal learning test, *Rey* Rey-Osterrieth complex figure test. (DOC 495 kb)

## Abbreviations

ACME: Average causal mediation effect
AD: Alzheimer’s disease
ADE: Average direct effect
AIC: Akaike information criterion
apoE: Apolipoprotein E
Aβ: Amyloid-beta
CSF: Cerebrospinal fluid
FDR: False discovery rate
FSIQ: Full-scale intelligence quotient
MCI: Mild cognitive impairment
MMSE: Mini mental state examination
MRI: Magnetic resonance imaging
NFT: Neurofibrillary tangles
PET: Positron emission tomography
RAVL: Rey auditory verbal learning
Rey: Rey-Osterrieth complex figure
ROI: Region of interest
SUVR: Standardized uptake value ratio
