# Supplementary material for: Regional tau deposition measured by [18F]THK5317 positron emission tomography is associated to cognition via glucose metabolism in Alzheimer’s disease
Source: Alzheimers Res Ther. 2016 Sep 29;8:38. doi: 10.1186/s13195-016-0204-z (PMC5041516; doi:10.1186/s13195-016-0204-z)
Supplement: Additional file 1: — Association of [18F]THK5317 distribution volume ratio (DVR) and [18F]FDG standardized uptake value ratio (SUVR) regional retention with cognitive performance, as determined by linear regression modeling, after adjusting for delay between assessments. Only significant p values (<0.05) are displayed. Values in bold indicate associations that survived false discovery rate (FDR) correction for multiple comparisons. β standardized β coefficient, FSIQ full-scale intelligence quotient, MMSE mini mental state examination, RAVL Rey auditory verbal learning test, Rey Rey-Osterrieth complex figure test. (DOC 186 kb) [file 13195_2016_204_MOESM1_ESM.doc]

**Additional File 1**: Association of [^18^F]THK5317 distribution volume ratio (DVR) and [^18^F]FDG standardized uptake value ratio (SUVR) regional retention with cognitive performance, as determined by linear regression modeling, after adjusting for delay between assessments. Only significant p-values (<0.05) are displayed. Values in bold indicate associations that survived false discovery rate (FDR) correction for multiple comparisons. β = standardized β coefficient; FSIQ = full-scale intelligence quotient; MMSE = mini mental state examination; RAVL = Rey auditory verbal learning test; Rey = Rey-Osterrieth complex figure test.

| **[^18^F]THK5317 DVR regional retention** | | |  | **Global cognition** | | **Episodic memory** | | |
| --- | --- | --- | --- | --- | --- | --- | --- | --- |
|  |  |  |  | **FSIQ** | **MMSE** | **RAVL, learning** | **RAVL, delayed recall** | **Rey, delayed recall** |
| **Temporal** | Amygdala |  | β |  |  | -0.572 |  |  |
| **cortex** |  |  | p |  |  | 0.012 |  |  |
|  | Fusiform | anterior  part | β | -0.546 |  |  |  |  |
|  |  |  | p | 0.023 |  |  |  |  |
|  |  | posterior | β | -0.657 |  |  |  |  |
|  |  | part | p | 0.005 |  |  |  |  |
|  | Parahippocampal | posterior | β | -0.650 | -0.518 | -0.547 |  |  |
|  | Gyrus | part | p | 0.003 | 0.027 | 0.019 |  |  |
|  |  | anterior | β |  |  | -0.690 |  |  |
|  |  | part | p |  |  | 0.002 |  |  |
|  | Inferior Temporal | temporo- | β | **-0.875** |  |  |  | -0.576 |
|  | Gyrus | occipital part | p | **< 0.001** |  |  |  | 0.024 |
|  |  | posterior | β | **-0.842** | -0.540 | -0.545 |  |  |
|  |  | part | p | **< 0.001** | 0.031 | 0.029 |  |  |
|  |  | anterior | β | -0.639 |  |  |  |  |
|  |  | part | p | 0.005 |  |  |  |  |
|  | Middle Temporal | temporo- | β | -0.688 |  | -0.565 |  | -0.571 |
|  | Gyrus | occipital part | p | 0.002 |  | 0.016 |  | 0.018 |
|  |  | posterior | β | -0.681 |  | -0.575 |  |  |
|  |  | part | p | 0.002 |  | 0.013 |  |  |
|  |  | anterior | β | -0.527 |  |  |  |  |
|  |  | part | p | 0.021 |  |  |  |  |
|  | Superior Temporal | posterior | β | -0.536 |  | -0.537 |  |  |
|  | Gyrus | part | p | 0.017 |  | 0.018 |  |  |
| **Occipital** | Occipital fusiform | | β | -0.606 |  |  |  | -0.518 |
| **cortex** |  |  | p | 0.007 |  |  |  | 0.032 |
|  | Lateral Occipital | inferior | β | -0.697 |  |  |  | -0.684 |
|  | Gyrus | part | p | 0.001 |  |  |  | 0.003 |
|  |  | superior | β | -0.544 |  |  |  | -0.614 |
|  |  | part | p | 0.023 |  |  |  | 0.012 |
|  | Angular Gyrus |  | β | -0.511 |  | -0.503 |  | -0.504 |
|  |  |  | p | 0.031 |  | 0.036 |  | 0.041 |
| **Frontal** | Inferior Frontal | pars | β |  |  | -0.580 |  |  |
| **cortex** | Gyrus | triangularis | p |  |  | 0.011 |  |  |
|  | Middle Frontal Gyrus | | β | -0.562 |  | -0.554 |  |  |
|  |  |  | p | 0.014 |  | 0.017 |  |  |
| **Parietal** | Precuneus |  | β | -0.638 |  |  |  | -0.538 |
| **cortex** |  |  | p | 0.004 |  |  |  | 0.026 |
|  | Parietal operculum |  | β | -0.471 |  |  |  |  |
|  |  |  | p | 0.045 |  |  |  |  |
|  | Cingulate | posterior | β | -0.561 |  |  |  |  |
|  | Gyrus | part | p | 0.013 |  |  |  |  |
|  | Supramarginal | anterior | β |  |  |  |  |  |
|  | Gyrus | part | p |  |  |  |  |  |
|  | Superior Parietal Lobule | | β |  |  |  |  | -0.512 |
|  |  |  | p |  |  |  |  | 0.030 |

| **[^18^F]FDG SUVR regional uptake** | | |  | **Global cognition** | | **Episodic memory** | | |
| --- | --- | --- | --- | --- | --- | --- | --- | --- |
|  | | |  | **FSIQ** | **MMSE** | **RAVL, learning** | **RAVL, delayed recall** | **Rey, delayed recall** |
| **Temporal** | Hippocampus |  | β | 0.541 | 0.603 | 0.532 |  | 0.563 |
| **cortex** |  |  | p | 0.029 | 0.011 | 0.019 |  | 0.028 |
|  | Planum temporale |  | β | 0.488 |  |  |  |  |
|  |  |  | p | 0.033 |  |  |  |  |
|  | Fusiform | temporo-  occipital | β | 0.605 |  |  |  |  |
|  |  |  | p | 0.006 |  |  |  |  |
|  |  | posterior | β | 0.574 | 0.469 |  |  |  |
|  |  | part | p | 0.012 | 0.042 |  |  |  |
|  | Parahippocampal | posterior | β | 0.465 |  |  |  |  |
|  | Gyrus | part | p | 0.044 |  |  |  |  |
|  |  | anterior | β |  | 0.581 | 0.527 | 0.523 |  |
|  |  | part | p |  | 0.009 | 0.020 | 0.021 |  |
|  | Inferior Temporal | temporo- | β | **0.782** | 0.574 |  |  |  |
|  | Gyrus | occipital part | p | **<0.001** | 0.011 |  |  |  |
|  |  | posterior | β | **0.813** | **0.725** |  |  |  |
|  |  | part | p | **<0.001** | **0.001** |  |  |  |
|  |  | anterior | β | 0.601 | 0.598 |  |  |  |
|  |  | part | p | 0.009 | 0.007 |  |  |  |
|  | Middle Temporal | temporo- | β | **0.779** | 0.547 |  |  |  |
|  | Gyrus | occipital part | p | **<0.001** | 0.018 |  |  |  |
|  |  | posterior | β | **0.823** | **0.651** |  |  |  |
|  |  | part | p | **<0.001** | **0.003** |  |  |  |
|  |  | anterior | β | **0.760** | **0.659** |  |  |  |
|  |  | part | p | **0.001** | **0.003** |  |  |  |
|  | Superior Temporal | posterior | β | 0.604 |  |  |  |  |
|  | Gyrus | part | p | 0.006 |  |  |  |  |
|  | Temporal Pole |  | β |  | 0.528 |  |  |  |
|  |  |  | p |  | 0.021 |  |  |  |
| **Occipital** | Occipital fusiform | | β | 0.504 |  |  |  |  |
| **cortex** |  |  | p | 0.027 |  |  |  |  |
|  | Lateral Occipital | inferior | β | 0.630 |  |  |  |  |
|  |  | part | p | 0.004 |  |  |  |  |
|  |  | superior | β | **0.815** | 0.588 |  |  |  |
|  |  | part | p | **<0.001** | 0.008 |  |  |  |
|  | Angular Gyrus |  | β | **0.869** | **0.698** | 0.577 |  |  |
|  |  |  | p | **<0.001** | **0.002** | 0.047 |  |  |
| **Frontal** | Inferior Frontal | pars | β | 0.545 | 0.533 |  |  |  |
| **cortex** | Gyrus | opercularis | p | 0.016 | 0.015 |  |  |  |
|  |  | pars | β | 0.489 | 0.520 |  |  |  |
|  |  | triangularis | p | 0.035 | 0.020 |  |  |  |
|  | Middle Frontal Gyrus | | β | **0.690** | 0.620 |  |  |  |
|  |  |  | p | **0.002** | 0.005 |  |  |  |
|  | Superior Frontal Gyrus | | β | 0.588 |  |  |  |  |
|  |  |  | p | 0.009 |  |  |  |  |
|  | Frontal Pole |  | β | 0.544 | 0.583 |  |  |  |
|  |  |  | p | 0.017 | 0.007 |  |  |  |
|  | Orbito-frontal gyrus |  | β |  | 0.460 |  |  |  |
|  |  |  | p |  | 0.044 |  |  |  |
| **Parietal** | Parietal Operculum | | β | 0.549 |  |  |  |  |
| **cortex** |  |  | p | 0.015 |  |  |  |  |
|  | Precuneus |  | β | **0.807** | 0.567 |  |  |  |
|  |  |  | p | **<0.001** | 0.010 |  |  |  |
|  | Cingulate | posterior | β | **0.766** | 0.549 |  |  |  |
|  | Gyrus | part | p | **<0.001** | 0.012 |  |  |  |
|  | Supramarginal | posterior | β | **0.858** | **0.648** |  |  |  |
|  | Gyrus | part | p | **<0.001** | **0.003** |  |  |  |
|  |  | anterior | β | **0.719** | 0.522 |  |  |  |
|  |  | part | p | **0.001** | 0.021 |  |  |  |
|  | Superior Parietal |  | β | **0.820** | 0.569 | 0.501 |  |  |
|  | Lobule |  | p | **<0.001** | 0.011 | 0.029 |  |  |
|  | Postcentral |  | β | 0.563 |  |  |  |  |
|  | Gyrus |  | p | 0.012 |  |  |  |  |
| **Insular** |  |  | β | 0.458 | 0.500 |  |  |  |
| **cortex** |  |  | p | 0.048 | 0.025 |  |  |  |
