# Supplementary material for: Regional tau deposition measured by [18F]THK5317 positron emission tomography is associated to cognition via glucose metabolism in Alzheimer’s disease
Source: Alzheimers Res Ther. 2016 Sep 29;8:38. doi: 10.1186/s13195-016-0204-z (PMC5041516; doi:10.1186/s13195-016-0204-z)
Supplement: Additional file 2: — Cortical regions of interest where PET tracer retention was significantly associated with cognition in AD patients, after adjusting for delay and age (not corrected for multiple comparisons). Linear models assessed the association between cognitive performance using five different tests (each row) and (A). [18F]THK5317 retention (DVR) or (B). [18F]FDG uptake (SUVR). Regions are defined according to the Harvard-Oxford atlas. T-values for significant negative associations are displayed with a blue color scale, and T-values for significant positive associations are displayed with a red color scale. FSIQ full-scale intelligence quotient, MMSE mini mental state examination, RAVL Rey auditory verbal learning test, Rey Rey-Osterrieth complex figure test. (DOC 495 kb) [file 13195_2016_204_MOESM2_ESM.doc]

**Additional File 2:** Cortical regions of interest where PET tracer retention was significantly associated with cognition in AD patients, after adjusting for delay and age (not corrected for multiple comparisons). Linear models assessed the association between cognitive performance using five different tests (each row) and A. [^18^F]THK5317 retention (DVR), or B. [^18^F]FDG uptake (SUVR). Regions are defined according to the Harvard-Oxford atlas. T-values for significant negative associations are displayed with a blue color scale, and T-values for significant positive associations are displayed with a red color scale. FSIQ = full-scale intelligence quotient; MMSE = mini mental state examination; RAVL = Rey auditory verbal learning test; Rey = Rey-Osterrieth complex figure test.

**
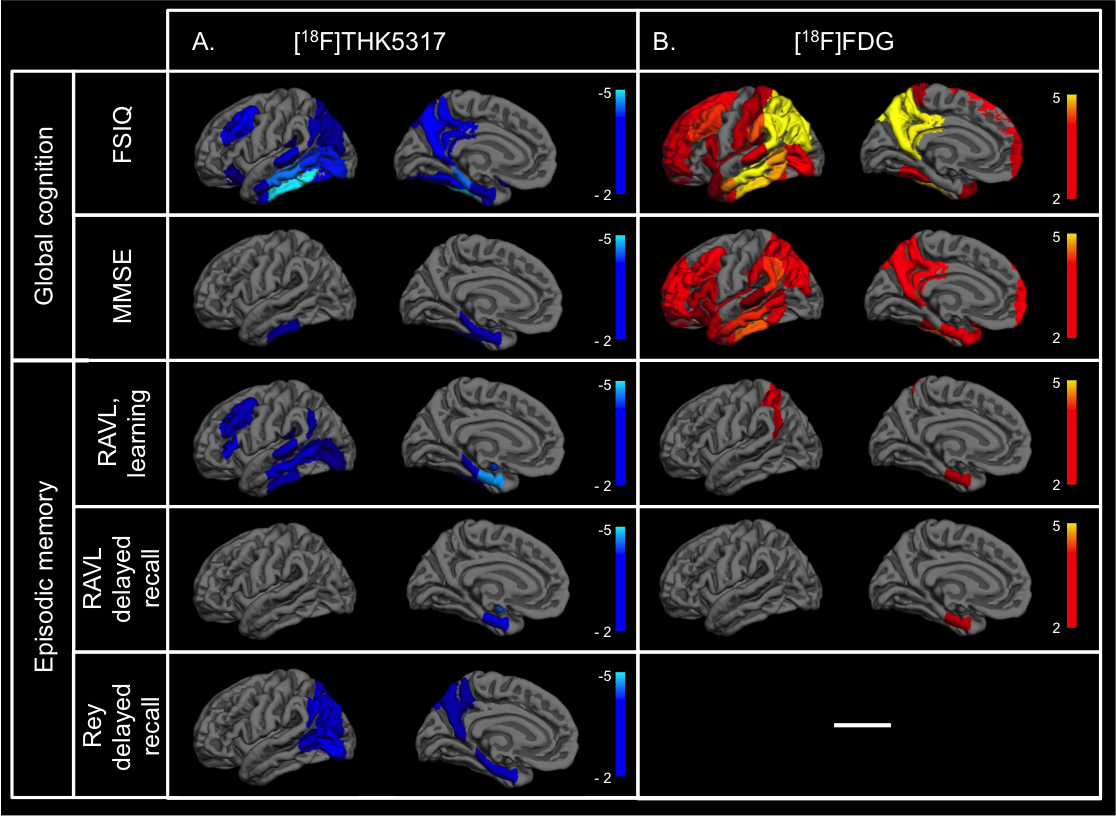
**
